# "The Hospital" Nursing Mirror

**Published:** 1897-06-19

**Authors:** 


### "The Hospital" Nursing Mirror.

Being the Nursing Section of "The Hospital."

[Contributions for this Section of "The Hospital" should be addressed to the Editor, The Hospital, 28 & 29, Southampton Street, Strand, London, W.C., and should have the word "Nursing" plainly written in left-hand top corner of the envelope.]

### News from the Nursing World.

### CANADA AND THE JUBILEE.

The National Council of Women of Canada have been holding their fourth annual session this week. Lord Aberdeen presided over a public meeting, at which a loyal address to the Queen was adopted, and speeches "full of feeling and patriotism" were delivered. The Council have re-elected Lady Aberdeen as their president.

### MUSIC HALLS AND THE JUBILEE.

A matinee in aid of the Queen's Jubilee Institute for Nurses is to be held at the Tivoli on the 28th instant, at which many great lights of the music-hall stage have volunteered to assist. Mr. J. W. Cragg, who presided over the meeting held to consider the matter at the Tivoli the other day, said that in his opinion it was only fitting that music-hall artistes should make some special effort to commemorate the illustrious reign of the Queen, and could do so in no better way than by supporting in some tangible form the fund on behalf of the Jubilee Institute.

### THE NURSE AND MATRON QUESTION.

The spirit in which Boards of Guardians too often meet the difficulties constantly occurring in country workhouses through the impossibilities of the trained nurse's position under the untrained matron was exemplified the other day at a Norfolk workhouse. There had been a complaint on the part of the matron respecting the nurse, and the House Committee reported to the board that they had gone into the matter, and had decided to give the nurse three months' notice. In spite of the desire expressed by some members of the board to hear for themselves the nurse's version of the troubles, and the pertinent suggestion of one guardian that "the nurse, a qualified person, did not like acting under an unqualified matron," the committee's report was accepted, the chairman saying that "the matron, without a doubt, must be the head." While the authority of the untrained matron is thus blindly upheld, merely because she *is* the matron, daily will it become more difficult to find nurses to fill the undesirable positions offered them in country unions, and the result can only be the gradual deterioration of the nursing in spite of all the efforts which have been made to raise and improve it. In the meantime, it may be asked, where are the new orders of the Local Government Board which are to do so much for workhouse nursing?

### RESIGNATION OF MISS LUMSDEN.

Miss Rachel Lumsden has resigned her appointment as honorary superintendent of the Aberdeen Royal Infirmary, a post which she has held for nearly twelve years. For twenty years past Miss Lumsden has worked in Aberdeen. She was largely instrumental in establishing the Hospital for Sick Children, to which, from its opening in 1877, she gave nine years' devoted service. In 1885 Miss Lumsden offered her services to the directors of the Royal Infirmary, and here she has remained to the present day in active work as honorary superintendent. At the earnest request of the Board she will remain at her post for a while longer to allow time for the appointment of a successor, and possibly to see the opening of the new surgical block. Miss Lumsden will be much missed and regretted, but all who know how hard she has worked during these years will feel that she has nobly earned a rest, and will wish her many years of health to enjoy it.

### NURSES IN BELFAST.

The Lady Mayoress of Belfast, in presiding over the annual meeting of the Society for Providing Nurses for the Sick Poor, reminded her audience that the claims of this excellent association must not be forgotten in the multitude of charitable schemes which are just now before the public. The Countess of Shaftesbury, who was present, spoke warmly of the good work accomplished among the poor and suffering through the agency of the district nurses. It was announced that the executive committee have approved of a plan of federation with the Royal National Pension Fund for Nurses, by which on certain conditions pensions of £20 a year will be secured to nurses who remain in the service of the society until they are sixty. This is a wise step on the part of the committee, upon which they are to be congratulated.

### WORKING LADIES' GUILD.

The annual meeting of the Working Ladies' Guild, 251, Brompton Road, S.W., was held on Tuesday at 48, Belgrave Square, by the kind permission of Lady Susan Leslie Melville. Canon Pennefather, Lady Grant-Duff, and Sir Henry Cunningham, K.C.I.E., addressed the meeting. The guild has had a very successful year's work. Increased subscriptions and donations from City companies have enabled it to help more liberally the class for which it works—distressed ladies. £2,000 has been distributed in pensions, training and education fees, relief in times of sickness and need, during the year, and nearly £2,000 more for work done in the guild house or sold in commission. The guild has been organised for twenty years, and needs only larger means to extend its sphere of usefulness in helping a very needy and very suffering class of society.

### NURSING AT THE CAPE.

Miss Martha Miller, a trained nurse, has offered her services free for a year to the "Victoria Nurses' Institute," which is being started at Cape Town in celebration of the Jubilee, in order to give the organisation a good start. At the inaugural meeting Mrs. Goodenough presided, and Lady Sprigg, Lady de Villiers, Mrs. Rawson, and other well-known ladies were present. We wish the association all success.

### THE SOCIETY OF CHARTERED NURSES.

We have received the first annual report of the Society of Chartered Nurses, founded last year. The society is constituted on co-operative principles, each nurse receiving her earnings, less 7½ per cent., for defraying working expenses. The nurses are members of the Royal British Nurses' Association, and must all have passed through three years of hospital training. There are now 65 nurses on the roll, and 15 working on probation. The report states that the society is in a sound financial position, and practically self-supporting, having defrayed all its working expenses during the year, the audited accounts showing that the amount received from patients for the year has been £5,424 12s., with a net profit on the year's working of £30 6s. 4d. H.R.H. Princess Christian has consented to become president. There is a strong committee of medical men and matrons, amongst the former, Mr. Langton (chairman), Sir James Crichton Browne, Sir Dyce Duckworth, Mr. Herbert Allingham, Mr. Brudenell Carter, Mr. Mark Hovell, and Mr. Pearce Gould; while the matrons are Mrs. Coster, Miss Hull, Miss de Pledge, Miss Thorold, and Miss Wedgwood. The society is to be congratulated on having made so good a start.

### THE READING NURSING INSTITUTE.

At a meeting of the committee appointed to frame the constitution of the new district nursing institution at Reading, some discussion took place as to whether it should be upon benevolent or provident lines. Dr. Hurry strongly urged the latter, suggesting that the working classes of the borough should contribute small annual subscriptions of 1s. (or 2s. 6d. for a family), entitling them to the services of a nurse in times of sickness. In his opinion such a plan would work as well as a provident dispensary, and by encouraging thrift would help the working classes to help themselves instead of depending upon charity. Dr. Hurry's proposal did not meet with approval, and was lost by eleven votes to seven. A scheme drawn up by Mr. Furguson on the ordinary benevolent system was carried. By its provisions patients who are able to contribute something to the funds of the institution will be allowed to do so.

### THE PLAISTOW MATERNITY CHARITY.

A bazaar and sale of work in aid of the Plaistow Maternity Charity and District Nurses Home was opened last week at the Stratford Town Hall by Lady Selborne. Brisk business was done during the afternoon, which we hope will have brought Sister Katherine some substantial help for her good work in "London over the Border." Afterwards the Mayor of West Ham held a reception, at which a number of visitors were present.

### THE MIDWIVES' INSTITUTE.

The next course of lectures on midwifery and monthly nursing will begin on Monday, August 23rd, for the October L.O.S. examination. The course consists of eighteen lectures from F. R. Humphreys, Esq., L.R.C.P., including the requirements of the L.O.S., supplemented by nine lectures on monthly nursing by Mrs. Hewer. Thus the midwifery and monthly nursing are admirably and completely combined together in one course, the fee for the whole twenty-seven lectures being the extremely moderate one of £3 3s. As some of the pupils may not be trained nurses, classes will be held by an experienced midwife for going through those parts of the lectures which may be found difficult, the fee for these coaching classes being 15s. A single lecture may be attended on payment of 2s. 6d. All particulars may be had from the Secretary of the Institute, 12, Buckingham Street, Strand, and early application should be made by those pupils who also wish a vacancy reserved for them with a midwife for the practical work. The lectures and classes take place on Mondays and Thursdays.

### ROYAL HOSPITAL FOR INCURABLES, DONNYBROOK.

Last week a garden party was held in the picturesque grounds of the Royal Hospital for Incurables, Donnybrook, in honour of the Diamond Jubilee. Some two thousand invitations were issued by Miss Burt, the lady superintendent, and the number of acceptances was very large. The entire expenses of the entertainment were defrayed by Mr. William Fry, vice-chairman of the Board of Governors, ever a generous supporter of the hospital. Before the arrival of the guests, those of the inmates who were able to leave their beds sat down to an excellent dinner, also provided by Mr. Fry. After the garden party, Miss Burt received the lady superintendents and nurses of the principal Dublin hospitals at supper, the evening concluding with music and dancing. The weather was all that could be desired, and the festivities were carried through with entire success. The Managing Committee decided, at a meeting held early in the day, to increase the salary of the staff nurses, whose services are greatly valued, by £5 per year, in honour of the Jubilee, besides granting an extra week's holiday to the nursing staff, and arranging for the servants to have a whole day off duty on the 22nd inst.

### BRISTOL GUARDIANS AND THE DISTRICT NURSES' SOCIETY.

The Bristol District Nurses' Society recently applied to the Guardians for an increase in their annual subscription of 10 guineas, in consequence of the large number of visits made by the society's nurses to persons in receipt of parish relief. The committee appointed to consider the question found that during the first three months of the year these visits amounted to an average of 28 per day, and recommended that for the current year the subscription be increased to 30 guineas.

### SHORT ITEMS.

A District Nursing Association is being formed at Warrington, starting with a staff of three nurses, which it will be the aim of the committee to increase to six as soon as possible.—Mrs. Watson Armstrong presided over the recent annual meeting of the Rothbury Nursing Association. Nurse Hugil did good work during the past year, paying altogether 2,431 visits; her salary is to be increased from £80 to £85 per annum.—By the request of Dr. Whitcombe, the superintendent, the House Committee of the Birmingham City Asylum have granted a week's extra leave, or a week's pay, to the whole staff, in honour of the Diamond Jubilee.—Nurse McMann thinks readers of The Hospital will like to hear of her good fortune in winning an Ormonde bicycle at the Earl's Court Exhibition. She was given a coupon "to write an epigram on the great wheel," and was successful in gaining the prize. We are sure all nurses who, with Nurse McMann, rightly look upon bicycling as a "grand exercise," will congratulate her warmly.

### Pharmacy and Dispensing for Nurses.

By C. J. S. Thompson.

### XIV.—LOTIONS—LINIMENTS—PIGMENTS AND OLEATES—OINTMENTS.

A lotion is a liquid preparation used as an external application or wash to various parts of the body. An eye lotion is usually termed a collyrium. Solution of sub-acetate of lead is a very frequent ingredient in lotions, and when diluted distilled water should be used, otherwise the solution will turn opaque. It is often prescribed with tincture of opium, and a very ugly precipitate results on mixing the two liquids; but this may be prevented if a small quantity of glycerine be mixed with the lead solution before adding the tincture. Acetate of lead is sometimes ordered in combination with alum or sulphate of zinc, with the result that a copious precipitate of sulphate of lead is thrown down. Such precipitates should not be strained out unless specially ordered. Solid extracts, when met with in lotions, should be placed in a mortar and rubbed down with hot water until dissolved, and then strained. Insoluble bodies, such as oxide of zinc, should be reduced to very fine powder and well diffused throughout the liquid menstruum. Lotions should always be dispensed in coloured bottles and bear distinctive labels, to prevent them being mistaken for medicines intended for internal use.

### Liniments.

The liniment or embrocation is the name given to a liquid application to be rubbed over the surface of the body. They are usually composed of a mixture of oils, soaps, or spirituous preparations, simple or compound. The liniments met with in dispensing are usually those of the Pharmacopœia, or a mixture of the same, as in the following instance: ℞ Lin. belladonna, ʒi.; lin. chloroform co., ʒiv.; lin. saponis, ʒiv. Misce fiat linimentum. A liniment of this kind presents no difficulty, the ingredients being simply mixed together in a bottle.

Liniments are sometimes prescribed in the form of an emulsion or soap, as for example: ℞ Lin. camph., ℥iiss.; liq. ammon., ʒii.; tr. opii., ʒii. Misce. In dispensing the camphor liniment should first be placed in the bottle, and shaken well with the solution of ammonia until a creamy emulsion is formed. Then add the tincture of opium, and again shake until the whole is thoroughly mixed.

The following liniment is frequently met with: ℞ Spt. terebinth, ℥ii.; acid. acetic fort, ʒi.; ovi vitel, i.; aquam., ℥iii. Misce. Place the yolk of egg in a mortar and add the turpentine in small quantities, stirring well between each addition, until it is thoroughly emulsified. Next add the acetic acid, and finally the water, then transfer to the bottle and shake well. The liniment of potassium iodide with soap of the British Pharmacopœia is an example of a liniment with a soap base. It is made by dissolving curd soap in fine shavings in glycerine and water, over a water bath. The iodide of potassium is placed in a mortar and powdered, and the soap solution gradually added to it, the whole being well stirred until cold. Oil of lemon is finally added.

Liniments must always be dispensed in coloured bottles bearing a distinctive label, and marked "for external use only."

### Pigments and Oleates.

An application that is directed to be applied by means of a brush is usually termed a pigment, or paint. Spirit or glycerine generally form the base of such applications, having some active agent in solution. The glycerines of acids —carbolic, gallic, and tannic—also of alum, of the Pharmacopœia, may be taken as examples of preparations of this class, all of which are easily prepared. Glycerine of belladonna, which is very often prescribed, may be prepared by placing one ounce of extract of belladonna in a warm mortar and rubbing it down to a smooth paste with one drachm of hot water, then adding sufficient glycerine to produce two ounces.

There are certain solid bodies which, when rubbed together, become liquid, as, for instance, chloral hydrate and camphor, chloral hydrate and menthol, also chloral hydrate and thymol. A warm mortar aids the process. These compounds are frequently employed as pigments. Oleates are formed by the combination of oleic acid, and with most metallic oxides. The acid acts as a solvent, the result being a solution of oleate in an excess of oleic acid. Another form of oleate which occurs in the form of fine powder, is made by the double decomposition of a soluble metallic salt, as, for instance, sulphate of zinc and castile soap. Thus made, the oleates contain no free oleic acid, and are often prescribed for dusting purposes in certain skin diseases. Oleates are very readily absorbed by the skin, and are used as a medium for applying mercury, bismuth, copper, lead, and zinc. Oleate of mercury is prepared of varied strengths. It is made by gradually adding one part of yellow oxide of mercury to nineteen of oleic acid, in a mortar, and stirring constantly until the oxide is dissolved. A solution of 5 per cent. remains liquid when finished, but if over 10 per cent., it assumes a semi-solid consistency. Alkaloids, such as morphine and quinine, are soluble in oleic acid, but their salts are not.

The oleate of zinc in powder is prepared by the other process as follows: Dissolve 1 lb. of castile soap in 6 pints of boiling water, and in another 16 ounces of boiling water dissolve 7 ounces of sulphate of zinc; add this to the former solution, stir well, separate the water from the oleate floating on the top, and wash the latter with hot water till free from sulphate, then cool, dry, and reduce to fine powder.

### Ointments.

An ointment may be described as a semi-solid application, the basis of which consists of a fatty body, such as lard, soft paraffin, lanoline, or combination of wax and oil, &c.

Ointments are prepared by simple mixing on a suitable glazed slab with a flexible spatula, or rubbing together in a mortar; or the base may be melted over a water bath and added to the medicinal agents, the whole being well stirred until cool. The latter method is generally adopted when a solid substance has to be dealt with. It must first be reduced to fine powder in a mortar, and rendered free from grittiness. Should the powder be bulky, it may be rubbed into a smooth paste with a little almond oil before adding the base to it. When the active ingredient is an alkaloid, and there is only a small quantity of it, a solvent is generally used, which ensures its thorough distribution throughout the base. Thus, in the case of ointment of atropine of the Pharmacopœia: Take of atropine, 8 grains; rectified spirit, ½ fluid drachm; benzoated lard, 1 ounce; dissolve the atropine in the spirit, add the lard, and mix thoroughly. In this, and similar cases, the base, of course, is not melted; and so, in dispensing ointments, the student must be guided by the nature of the ingredients ordered as to the best means of combining them. Thymol should be heated in the melted base until dissolved, as undissolved particles are apt to produce irritation when applied to the skin. Iodoform, on the other hand, should not be heated, but incorporated with the base while it is cooling. When mixing glycerine with fats, the mortar used should be slightly warmed, and soft extracts should be rubbed down to a smooth paste with a few drops of hot water before the base is added.

An ointment, when finished, should be bland and smooth throughout, and free from lumps and grittiness.

### Post-Graduate Clinics for Nurses.

By a Trained Nurse.

XVIII.

I wound my article of last week to a close by opening up a consideration of the several points on which a nurse may have to unlearn in private practice many details of nursing which she had been accustomed to carry out in her training school. And I began a criticism on the practice common enough in hospitals of getting the convalescents out of bed about a quarter past one. Now this means that almost immediately after the principal solid meal of the day, just when the patient most needs rest to enable his weakened digestion to extract all the possible nourishment afforded by his dinner, he must needs be roused. In the hurrying and dressing, amid the natural excitement of a "first sitting up," every physiological law is thus broken, often without the least consciousness on the part of the nursing staff that this is so. In my own training school this hurried process of dressing after dinner was universal, and I never heard a word of protest raised by any of the "sisters"—who were, of course, the bulwarks on whose superior knowledge the nursing staff relied—against such a breakage of the fundamental principles of good nursing. And yet, in mentioning the matter since to nurses, they generally at once realise that all the traditions of good nursing are contravened by putting a convalescent through such excitement and muscular exertion when what he really needs after his dinner is a good hour and a-half's rest and sleep. After this he could go through the tiring process of dressing and sitting up without undue fatigue or excitement. And he would naturally enjoy the little change more, and look upon it as a pleasant, refreshing little incident, just because it took place at the right time.

"Of course your reasoning is perfectly right," said an intelligent sister, when spoken to on this subject. "But I could not have my ward in a perpetual muddle through having patients dressed after everything is 'tidied up' for the afternoon. I call that 'coddling,' not nursing, and I don't believe nurses would stay in a ward if they were 'fussed' about little things of that kind." To reasoning like this there is very little to be said. But, fortunately for the sick thousands in London and elsewhere, the great majority of nurses spare no pains to do that which they believe best for their patients. In private practice I have been surprised to find how many nurses keep to the tradition of their hospital from sheer force of habit, and induce their patients to get out of bed and sit up immediately after the mid-day meal.

Now in private nursing the same reason for such a practice does not exist as is supposed to exist in hospital. There is no uniformity of idea for a single sick case. The room belongs to him, there is not the stately round of the visiting physician and his retinue to be thought of, and there is no definite hour at which the patient and his apartment must be in apple-pie trim. In this case the nurse is simply following a habit she gained while training, and the sooner she loses such a habit the better for her advancement as a nurse, and the better for her patient's appetite, health, and digestion. Let the patient, after a meal, lie silent, reposeful, and quiet. In summer or in bright weather get him into the habit of having his blinds drawn down, so that in a dreamy semi-darkness, with a soothing silence around him, he may digest his food—and sleep awhile. Thus will a sick person under the *régime* of a wise nurse flourish like a green bay-tree!

There are several other little salient points connected with the influence of rest on digestion which it is well for the nurse to realise, for the consideration of these will serve her in good stead in the many cases of illness which are complicated with digestive trouble. Perhaps next in popularity to the custom of "getting patients up" immediately after a meal is the habit of getting them up to a meal. And this is frequently done in some cases of marked digestive trouble, or in patients whose illness is accompanied by much prostration and weakness.

The excitement and exertion consequent on "getting dressed" is very apt in such cases to interfere with the meal, especially as the very fact of "sitting to a table" after being long accustomed to take food lying down will often tempt the patient to mark the festivity by a greater indulgence than usual in the good things of this material life. Experience will of course show whether getting up to a meal causes digestive discomfort; but in those cases where the digestion is known to be weak, the nurse should take it as a foregone conclusion that the stomach will not accomplish its work so well after the exertion of dressing.

It takes many years to find out for oneself by experience the thousand and one little subtleties of nursing—those "things which matter" so much and add up to such a huge sum in the recovery of a sick person. A belief lives strong in the hearts and minds of the majority of mankind that a quick and brisk walk "gets up an appetite," and enables a tired pedestrian to digest a hearty meal. There are healthy, robust persons who find by experience that strong exercise taken before a meal has little or no effect on their digestive capacities. But such persons will not need to call the nurse into requisition. With invalids the reverse as to appetite and exercise is true. The exercise is good, if taken at the right time, but when I see—as I frequently do—whole generations of nurses at Bournemouth, Ventnor, and elsewhere, proudly taking out the sick, the prostrated, and the consumptive, for "a nice walk before luncheon—just to get up an appetite"—I feel inclined there and then to stand up on the parade and hold an open-air protest against so unhygienic a method of procedure. And I feel on such occasions that I would give half my possessions to carry a banner emblazoned in letters of gold with such inscriptions as these: "After a walk the invalid or convalescent should lie down for at least half an hour before taking a meal." "Exercise disperses the blood through the body. For the digestion of food the blood is needed in the stomach."

I am quite prepared to admit that it would need much courage to carry such a banner through the streets, but in the interests of the whole invalid family I think such a crusade would be worth organising. It is common enough to hear invalids who have, themselves, as well as their nurses, a full and firm faith that exercise must needs effectually whip up their digestive machinery to good service, complain "My luncheon did not agree with me. I have had such a sensation of heaviness all the afternoon. And yet I took such a good walk this morning and had my food directly afterwards." But that was just the fatal error. The exercise would no doubt have acted as a splendid tonic taken at the right season. And the vital forces fortified by a pleasant stroll in the sunshine and fresh air would have furnished fresh energy to the stomach. But in order to concentrate the vital energy towards the stomach after the expenditure of force consequent on a walk, it is necessary to lie down and rest for awhile. The blood which by exercise has been distributed through the entire frame—to the great benefit of the general health—will thus have time, as it were, to settle down to the normal, so that when the food be eaten a proper supply of blood and energy will be available in the stomach for the efficient digestion of such food. For this simple reason dyspeptics of many years' standing find a ready cure for their trouble by an invariable rule of lying down for half an hour before each meal.

### The Old Hospital of the Knights at Malta.

(FROM A CORRESPONDENT AT MALTA.)

THE Order of the Knights of St. John established themselves in Malta in 1530, and first nursed their sick at Citta Vechia, the old capital town of the island, a transfer being afterwards made to Borgo, or Vittoriosa as it is now called, as a more convenient site for a hospital, but in 1575 they built their large infirmary in the new city of Valetta. This hospital was arranged to accommodate between 700 and 800 patients, and had one tremendous ward 503 ft. long by 34 ft. wide and 30 ft. 11 in. high, with another in the basement of the same size, and various smaller wards, kitchens, and rooms for the officials, of which body there were a great number. The walls were extremely thick, as one can see at the present time, and judging from a quaint old print of the big ward daylight and sunshine were not valued as they are nowadays; the picture shows few windows and those small and very high up. The site of the hospital, just above the Grand Harbour, was chosen with a view to its being an easy spot at which to land the sick. One wonders where all the patients came from to fill the 400 beds, which was the average number occupied. Malta in those days would hardly supply such a number of invalids, so the Knights must have brought them from other countries, especially as we find it mentioned that men of all degrees and faiths were treated in the hospital. The Knights had 24 beds reserved for themselves, and their slaves—of whom the Order possessed large numbers—occupied the big ward in the basement. A gloomy place it must have been.

The old picture referred to above represents all the beds with curtains, some of the four-poster arrangement and others in the form of bell tents. These were used to keep away the mosquitos and flies, which were sure to have been even more troublesome in the pre-antiseptic age than now. The supply of personal and bed linen was generous, and in the rules framed for the proper conduct of the work of the hospital special instructions were given for the frequent making and changing of the beds. All the linen was kept in a large store called the camerata, facing the entrance to the hospital, and now used as quarters for married soldiers and their families. All the patients—except the slaves, who had pewter vessels—were served on silver, and their diet was plentiful and varied; so varied, in fact, that one smiles to think of sick people being given "sweetmeats of two kinds," which are described as being of a sticky nature and mixed with almonds, evidently a form of the famous Maltese nougât of the present day.

The spiritual needs of the patients received as much attention as their temporal requirements, and altars were erected in every ward in addition to the chapel for the general use of the hospital. The chaplains numbered ten. The dying were placed in a small ward close by the chapel, so that they might join in the services.

The official head of the hospital was the Grand Master himself; next to him came the Grand Hospitaller, chief of the French Largue, or community; then the actual manager of the hospital, the infirmarian, a professed Knight, who held the post for two years, resided in the building, and was responsible for the welfare of the sick in every detail, the good conduct and discipline of all the officials in their various departments, and had also to supervise the physicians when treating their patients. The infirmarian had two assistants, "professed knights of integrity," called prodomi; their duties were to see that all allowances were correct in quality and quantity, to distribute medicines and food, and to keep accounts. There were various outside charities, such as the Foundling Hospital (La Falanga) and numerous doles for the poor, that the infirmarian and his two subordinates had also to superintend. The prodomi were allowed a secretary between them. The armoriere was an individual in whose care was the silver plate of the hospital, and who was responsible for its safety and cleanliness; then came the "clerk of the habit," who had an easy berth, consisting only of registering the expenses in the principal ledger, being present at distribution of doles, and drawing up the wills of the sick. The work of the linciere was of a more arduous nature, as he had charge of the linen, furniture, and bedding, but he was allowed a servant and a couple of slaves to cleanse, repair, and remake bedding. The botigliere looked after the stores of wine, oil, and bread; he also had an assistant. In addition to this list of three chief and four subordinate officers, there were two cooks, one purveyor (?), fourteen servants called warders—whose duty it was to attend the sick night and day—and two doorkeepers. The cooks and warders had forty-four slaves between them, drawn from the prison, to help in the work. Most of the officials resided in the hospital.

There were three principal physicians and the same number of surgeons, who took it in turns to be on duty, a month at a time, during which period they lived in the hospital, and visited the sick twice a day. There were also two assistant physicians, and surgeons, and to help the latter six young men called barbarotti who acted as dressers. In addition to these was a phlebotomist and two assistants. The Knights also paid a physician to give a daily public lecture on anatomy. The only female we hear of was a woman described as "elderly and experienced," who, however, lived apart from the hospital and attended to scurvy cases.

There were six officials in the dispensary, one chief and five assistants. Their duties must have been very heavy, for, besides making up the medicines for the hospital and all the various charities supported by the Knights, they had to supply the needs of any religious house, pilgrim, or poor person who asked for drugs; then they also dispensed milk, soup and restoratives to destitute persons with orders from the authorities.

The ecclesiastical staff of the hospital consisted of the prior, the vice-prior, eight priests, and a sacristan; a priest of the Greek Church also paid periodical visits. The duties of the prior and his vice were entirely spiritual, but the eight assistants had often to perform work which at the present would only be done by a nurse, such as feeding helpless cases and watching the dying, while the sacristan was expected to follow the doctors round the wards. The Brothers of the Order came to the hospital for instruction in first aid and nursing, and on Holy Thursday the Grand Hospitaller of the French Langue and all the French Knights came and "with exemplary charity" washed the feet of twelve poor men.

The rules of the hospital were modified from time to time, as occasion arose, and additions were made to the building for the same reason. Perellos seems to have done more in the building line than any other Grand Master, as he constructed the present front of the hospital with the principal entrance facing the street, besides spending £722 in general repairs.

The following is an extract from a quaint record of a visit paid by the chaplain of H.M.S. "Bristol" in August, 1676: "The hospital is a vast structure, wherein their sick and wounded lye. 'Tis so broad that twelve men may walk with ease a brest up the midst of it, and the beds are on each syd, standing on four yron pillars, with white curtains and vallends and coverings extreamly neate, and kept clean and sweete; the sick served all on silver plate; and it contains above 200 bedds below, besydes many spatious roomes in other quadrangles for the chiefe cavaliers and knights, with pleasant walkes and gardens, and a stately house for the chiefe doctor and other his attendants."

John Howard visited Malta in 1786, and made a searching inspection of the hospital, his impressions being quite different from those of the chaplain of the "Bristol." Perhaps during the period that elapsed between their visits the management had fallen into incapable and careless hands, or the experienced eye of John Howard may have detected faults where a superficial observer would find none. At any rate, he says, in his "Lazarettos of Europe," describing the hospital in 1786: "The pavement is of neat marble (or stone) squares. . . . All the patients lie single. One ward is for patients dangerously sick or dying, another for patients of the middle rank of life, and the third for the lower and poorer patients. In this last ward, which is the largest, there were four rows of beds, in the others only two. They were all so dirty and offensive as to create the necessity of perfuming them. . . . The use of perfume I always reckon a proof of inattention to cleanliness and airiness, and this inattention struck me forcible on opening some of the private closets, with which the ward is very properly provided. . . . Patients are twice a day served with provisions . . . broth, rice soup, and vermecelli are brought in dirty kettles, first to the upper hall, and poured into three silver bowls, out of which the patients are served. . . . I objected to the sweet cakes, and two sorts of clammy sweetmeats which were given to the patients. . . . These (the patients) were served by the most dirty, ragged, inhuman persons I ever saw. I once found eight or nine of them highly entertained with a delirious dying patient."

The great philanthropist found nothing to praise all through the hospital, the management of which must have become woefully lax since the noble commencement in 1575. He expressed his sentiments freely to the Grand Master, giving offence thereby, but his words bore some fruit in the shape of certain improvements introduced after his visit.

A few years after this the downfall of the Order began, and their ruin was completed in 1798, on the French invasion of Malta under Bonaparte.

The old building is practically as sound as ever, and has been in use for a number of years as a military hospital for English troops stationed at Valetta, and has 212 beds. The Malta Artillery have their hospital within its walls with 20 beds, and a distinct medical staff; and there is also a comfortable little hospital for soldiers' wives, and quarters for officers, nursing sisters, and men of the Medical Staff Corps.

The venerable pile still retains its picturesque appearance in spite of all the needful changes worked by modern science, and on moonlight nights it is not at all difficult to conjure up the black mantle of a knight with its white cross flitting across the quadrangle or past the open arches of the corridors.

Reference has been made for many of the above details of the work carried on by the Knights of St. John to a translation by the Rev. W. Bedford of an old Italian work preserved in the archives of Malta.

### Appointments.

MATRONS.

COUNTY INFIRMARY, CARLOW.—Miss F. C. Leonard has been appointed Lady Superintendent of the County Infirmary, Carlow. Miss Leonard was trained in the City of Dublin Hospital, and afterwards was appointed charge nurse at the Newport and Monmouthshire Infirmary. She then held the post of night superintendent at the Royal Hospital, Belfast, which post she leaves to take up her new duties in Carlow.

CONVALESCENT AND SURGICAL HOME FOR BOYS, BANSTEAD.—Miss Ida Chinnery has been appointed Matron at this institution. Miss Chinnery trained at the Middlesex Hospital, and has since worked for seven years at the Cama Hospital, Bombay.

### Hospital Etiquette and Discipline.

BY A MATRON.

THERE are two points which are invariably matters of surprise to the new probationer. She may have theorised beforehand, but it is only upon her entrance into hospital life that she practically realises either the one or the other. Both are so different to what she has been accustomed in her home, where generally there are but two social orders—the family and the servants. It comes as an astonishment to find the various grades of officialism in hospital, and to be obliged to discern the deference due to each.

The first thing to be done is to abolish all idea of social pre-eminence. This, as a rule, does not exist apart from professional distinction, and there are few hospitals now where the treatment of the lady or special probationers differs from that of the ordinary nurse-probationer, and this is as it should be. No quality or accomplishment need lie dormant in a nurse's career. There is full scope for every talent, and education and superior mental calibre will always bring preferment eventually. Everyone will rise to their proper level, but all must do so from the bottom of the gauge.

To a great extent it is the observance or neglect of the little conventionalities to which I have alluded that gives the tone to a hospital or ward. The sisters are mainly responsible for the inculcation, both by example and precept, of this special code of manners. From their demeanour to the medical staff, to the matron, and to each other, their subordinates will take their cue, and a sister who withholds respect will not receive it. It should be part of her duty to educate her nurses and probationers to be officially polite, looking upon it as an essential item of their training. In these democratic days we are apt to let slip distinctions of all sorts, but good tone and high moral influence wane in consequence.

Hand-in-hand with good manners runs strict discipline. It is the basis upon which the training is built, and yet it is a point many fail to understand. If those in highest authority are lax, the entire hospital suffers; if sisters are slack their wards become inefficient; if the nurses are not in earnest the probationers at once become demoralised. It does not do to relax the strict laws of discipline. The time comes when we appreciate the value of the training in the wards where we worked the hardest, where we learnt the most, and where the sister was a strict disciplinarian.

If it is hard sometimes to be "called over the coals," to be subjected to correction and made to keep rules, it is far more irksome to those who have to point out the rules and to administer rebuke. But those who have to train others must not be wearied by the monotony of the work, nor be tempted to relinquish the constant reiteration of well-worn axioms, and the tedious task of enforcing rules—for such is their duty. And those who seek to be trained must bear in mind that the process is essential for their ultimate success, and try to believe that they will one day be wise enough to be grateful for what at the time has been tiresome and vexatious.

### Where to Go.

THE MATERNITY CHARITY AND DISTRICT NURSES' HOME, PLAISTOW, E.—The fourth annual meeting of this charity will be held at Stafford House on Thursday, July 8th, 1897, at half-past three p.m., by kind permission of the Duke of Sutherland. The Duchess of Sutherland has consented to preside. The names of speakers will be announced in the *Times* and *Morning Post* on or about Monday, July 5th.

TRAINED NURSES' CLUB, 12, BUCKINGHAM STREET, STRAND, W.C.—Nurses are reminded that this club will be open throughout Jubilee Day, and that they may make use of the rooms for a "wash and brush up" and resting between the procession and illuminations for the small charge of 1s. Tea can also be obtained.

### Everybody's Opinion.

### NARROW-MINDED NURSES.

"One of Them" writes: I am afraid that continual nursing and the general work of hospitals, amidst which we see so much sickness, suffering, and sorrow, tends to make us a wee-bit narrow-minded; it is all in the same groove. Will our critic put forth a helping hand, and show and help us to emerge from its thraldom. It would be kinder than criticism.

### TRAINING FOR DISTRICT NURSES.

Miss K. S. Meyer, Superintendent North London Nursing Association for Providing Trained Nurses for the Sick Poor, 413, Holloway Road, N., writes: In answer to Query 280 in a recent issue of The Hospital Nursing Mirror, may I ask you kindly to state that at the North London Nursing Association, 413, Holloway Road, training in district nursing is given to nurses (being gentlewomen) who have been already fully trained in hospital. The work here is done under trained and experienced supervision, and the facilities for giving such training are remarkably good. This home was opened in 1877 as the first branch of the Metropolitan and National Nursing Association, and it is not in affiliation with the Queen's Jubilee Institute. For the last 20 years we have trained district nurses whether for our own staff or for work elsewhere. I trust that you will be so good as to insert this information in your next number.

### NURSES' HOME OF REST.

"A Cottage Hospital Matron" writes: Many letters have appeared in your paper on the subject of founding a home of rest for nurses during this year, but no one seems ready to start the effort. I feel quite sure the money necessary would be forthcoming if we could get some well-known men of business capacity interested in the matter to act as trustees, and I am writing to ask if you can help us in this matter by inducing some such to take the matter up, or by suggesting some gentlemen to whom we could write in the first instance. I feel quite sure we should have your kindly sympathy and the help of your valuable paper, and I hope also to have your advice as to the best basis on which to found such a home and the best means of setting to work. If I am not trespassing too far on your time, I should like to give you, in outline, my own idea, that you may tell me if you consider it practicable or no. A small house (capable of enlargement) to be built, with, say, six small bed-sitting-rooms, a common dining, and one other reception-room, kitchen, &c., at about £600 or £700; three small rooms to be endowed at, say, £500 each, and three let to nurses who have small incomes at from 7s. 6d. to 10s. a week. Donors or collectors of £500 to act on committee of management, and have right to keep one room occupied by permanent resident or by nurses needing rest from time to time. Rooms endowed by numerous small subscriptions to be allotted by committee, they having before them the needs and claims of each applicant. I think it would be a pity to attempt making the home self-supporting, as this must of necessity exclude from its benefits all those nurses whose income is too small to make adequate provision for old age, and it is, of course, these we mostly wish to help. I may say I am not personally likely to need the benefits of such a home, but like many another nurse should be only too glad of an effectual way to help my less fortunate sisters to a happier eventide. Do you think we could get advice or help through the Nurses' Pension Fund Committee or from Her Royal Highness the President herself?

### Presentations.

The Committee of the Chelmsford Infirmary have presented the Matron, Miss New, who has left to be married, with a handsome kettle and spirit stand for afternoon tea, bearing a suitable inscription, expressing their appreciation of her valuable services for the last fifteen years; also a purse containing a cheque. A silver-mounted crystal scent bottle has also been presented to Miss New by the ladies of the Samaritan Working Party, who hold their meetings at the infirmary.

### "The Hospital" Convalescent Fund.

### THE NURSES' BED.

When our readers first assisted us to raise a fund which had for its object the provision of a free bed at the seaside for convalescent or tired nurses, it was considered advantageous for it to be permanently established on the South Coast. Experience has since shown that such an arrangement had disadvantages, invalids in the North of England being debarred by distance from using the bed. Therefore it was decided that nurses helped by the fund should be sent to some seaside or country place within a moderate distance of their work to save them needless expense and fatigue. As the fund is utilised only for nurses whose own means are insufficient to give them necessary change after illness, or when they are run down, the expenses of long journeys are obviously impossible for themselves, and undesirable for The Hospital Convalescent Fund.

A good many nurses have benefited much by such rest and change—some who had broken down after heavy work, or who needed a stay at the seaside after operation or serious illness. In most cases a month's stay is arranged for, and Brighton, Littlehampton, Worthing, and Ventnor have been chiefly the places chosen, in every case encouraging reports being received from the nurses of the kindness and care they have met with. The last case dealt with was that of the nurse for whom an appeal was put in these columns quite lately, who wanted help to enable her to go out to a relation in America for a year's holiday. The Convalescent Fund headed the subscription list with £2, and, in all, £7 was handed over to Nurse K., who started on her journey early this month.

We do not trouble our readers with petitions for assistance while we have funds in hand sufficient for emergencies; but we do not want to let the fund drop below a certain point, and should now be glad of a little further help. The balance stands at £11 6s. 8d. at the present moment, a sum which we should like to augment before the end of the summer.

All contributions to the fund are acknowledged in The Hospital, and should be sent to the Honorary Secretary of the Fund, 28 and 29, Southampton Street, Strand.

Applications for assistance are not made public, and should be addressed to the Hon. Secretary, accompanied by a reference from a doctor and one from a matron or other responsible person stating the kind of change needed, for how long, and if it is likely to be of permanent benefit to the nurse.

We gratefully acknowledge the receipt of 2s. 6d. from Miss Dinwoodie.

### The Victoria Commemoration Club.

The arrangements made for the Jubilee Day at this club for nurses are likely to be of great service to members and their friends, and especially to country members. It will be a most difficult matter to procure refreshments of any kind on Jubilee Day, and fortunate are those nurses who have such a comfortable haven as a club to turn to. The Secretary of the Victoria Commemoration Club has provided a most tempting bill of fare for the days preceding and following June 22nd, as well as on the day itself, and it is to be hoped that members will give an early notice of their intention to make use of the resources of the club to the Secretary, that adequate provision may be made.

### Prizes for Nurses.

In our advertisement columns will be found a list of the fortunate winners of the prizes in the competition offered to nurses by Messrs. Southall Bros. and Barclay, of Birmingham, in commemoration of Her Majesty's Diamond Jubilee. We congratulate Miss Olivia Richards and the other nurses on their success. We have often had occasion to call attention in these columns to the excellence of the specialities manufactured by this firm, and we trust that their enterprise will meet with the success it deserves.

### For Reading to the Sick.

THE VALUE OF LITTLE THINGS.

**Verses.**

What can we do for Jesus' sake
Who is so high and good and great?
We know the Holy Innocents
Laid down for Him their infant life,
And Martyrs brave and patient Saints
Have stood for Him in fire and strife.

We wear the crown they wore of old,
Our lips have learned like vows to make;
We need not die, we cannot fight;
What may we do for Jesus' sake?

Oh, day by day, each Christian child
Has much to do, without, within;
A death to die for Jesus' sake,
A weary war to wage with sin.

When deep within our swelling hearts
The thoughts of pride and anger rise,
When bitter words are on our tongues,
And tears of passion in our eyes:

Then we may stay the angry blow,
Then we may check the hasty word,
Give gentle answers back again,
And fight a battle for our Lord.

With smiles of peace, and looks of love,
Light in our dwellings we may make,
Bid kind good humour brighten there,
And still do all for Jesus' sake.

There's not a child so small and weak,
But has his little cross to take,
His little work of love and praise
That he may do for Jesus' sake.

—*Hymns Ancient and Modern.*

**Reading.**

We ought to cherish the small virtues which grow at the foot of the Cross, for they are watered with the blood of the Son of God. These virtues are humility, patience, sweet temper, kindness, helpfulness to our neighbours, graciousness, good will, heartiness, sympathy, readiness to forgive, simplicity, truthfulness, and others like them. The virtues are like the violets, which love the coolness of the shade, which are fed with dew, and which, though they have no brilliancy, cease not to shed fragrance around. There are great virtues on the top of the Cross which have great splendour, especially when they are accompanied with love; such are wisdom, justice, zeal, liberality, and such-like. And everyone wishes to have these virtues because they are the most esteemed and make us the most thought of. But we should not judge of the greatness or littleness of a virtue by what it appears to the outward eye, for a virtue that is very small in appearance may be practised with great love to God, while one that is more shining may go along with very little love; yet this is the measure of their true value before God. —*S. Francis de Sales.*

There is no action so slight or so mean but it may be done to a great purpose and ennobled thereby, nor is any purpose so great but it may be helped by slight actions. . . . There is nothing so small but that we may honour God by asking His guidance of it, or insult Him by taking it into our own hands.—*Ruskin.*

### Minor Appointments.

St. Pancras Workhouse Female Insane Wards.—Miss Sophia Phœbe Bryant has been appointed Superintendent of these wards. Miss Bryant holds the certificate of the Medico-Psychological Association of Great Britain and Ireland, and trained for two and a half years at the Rainhill Asylum. She has also held appointments at Stockport Industrial School as sewing mistress and nurse, and at Caine Hill Asylum as charge nurse for three and a half-years.

### Notes and Queries.

**The contents of the Editor's Letter-box have now reached such unwieldy proportions that it has become necessary to establish a hard and fast rule regarding Answers to Correspondents. In future, all questions requiring replies will continue to be answered in this column without any fee. If an answer is required by letter, a fee of half-a-crown must be enclosed with the note containing the enquiry. We are always pleased to help our numerous correspondents to the fullest extent, and we can trust them to sympathise in the overwhelming amount of writing which makes the new rules a necessity. Every communication must be accompanied by the writer's name and address, otherwise it will receive no attention.**

*Aged Pilgrims' Friend Society.*

(290) Can you tell me the address of this society, of which I saw a notice in The Hospital recently?—*Nurse D.*

The address of the Aged Pilgrims' Friend Society is 83, Finsbury Pavement, E.C.

*A Probationer's Difficulty.*

(291) Please advise me. A probationer is advertised for at a salary of £18 per annum, and although the salary is to be paid monthly, nothing is said as to the period of engagement. Under these circumstances is there any period of engagement understood by custom?—*A. B.*

Every hospital and institution makes its own rules with regard to the length of training given to probationers. What do the printed rules of the institution in question say? We should be better able to advise you if you gave more detailed particulars, but if no agreement was signed we should think a month's notice on either side would be all that could be claimed. A probationer's object, however, is to gain a certificate, and a proper agreement should always be entered into.

*Nursing and Spelling.*

(292) Please tell me if it is necessary to be good at spelling to be accepted as a probationer? I have had an ordinary education, but am not a good speller. Where should I apply?—*Anxious to Know.*

A fairly good education is considered an essential qualification at most hospitals. Read "How to Become a Nurse" (Scientific Press, 28 and 29, Southampton Street, Strand), and apply at some of the hospitals and infirmaries, of which you will there find lists.

*Advice Wanted.*

(293) (1) Can you tell me who is the best specialist for brain and nerve disorders? I wish to take a friend to consult someone. She is suffering terribly from sleeplessness. (2) Is the "Nurses' Directory" out yet, and can you tell me of any place near Earl's Court where one could see a copy?

(1) We cannot recommend medical men. Advise your friend to consult a good general practitioner, and, if necessary, he will recommend a specialist. (2) The "Official Nursing Directory" is not yet published.

*Training.*

(294) I am completing a two years' course of surgical training, with certificate. Can you tell me where I can get a short medical training, either one or two years?—*Lucille.*

You will find it difficult to get a short term of training in purely medical nursing unless you are prepared to pay. See "How to Become a Nurse" (Scientific Press, 28 and 29, Southampton Street, Strand), where you will find particulars given of the length of training at London and provincial hospitals. Consult this list, and write to those hospitals which seem to suit your requirements best.

*Register or Case Book.*

(295) Please recommend.—*Enquirer.*

Write to Lowe Bros., 157, High Holborn, who can supply excellent case books of the kind you require.

*Dispensing.*

(296) Can you recommend dispensing as a lucrative and satisfactory profession for women? Can one learn the rudiments at home, or where could one best learn dispensary work?—*Dispensatorium.*

We cannot reply to queries unaccompanied by name and address. These are not for publication, but as evidence of good faith for the Editor's information.

*Nursing the Insane.*

(297) *Subscriber*, who asks about asylum training, is referred to the answer to *Dispensatorium* above.

*Daily Nursing.*

(298) Can you give me any information respecting nurses going out daily from their own homes by the hour or half-day to private houses where a resident nurse is not required? What are the fees usually charged, and is it necessary to be fully trained?

Write to Miss C. J. Wood, The Nurses' Hostel, 27, Percy Street, Tottenham Court Road, who has tried the daily nursing system from the hostel, and will be pleased to give you information. Daily nurses are much needed, but it would seem to be difficult, if not impossible, for one nurse, living at home, to make a financial success of the plan. It is desirable for three or more to join together. Miss Wood can tell you about fees. Certainly no nurse who is not fully trained should attempt private nursing. A nurse finds quite as many demands upon her skill and experience in daily nursing as in ordinary private nursing, and especially needs to be up to date in surgical work, attendance at operations being often required.

*Antiseptics for Nurses.*

(299) Can you tell of a book on antiseptic work suitable for a private nurse, and not too expensive?—*B. W.*

There is a 1s. book, "Antiseptic Principles for Nurses," published by J. and A. Churchill. A series of articles on the same subject will shortly be published in The Hospital.

